# Evaporation-Induced Transformations in Volatile Chemical Product-Derived Secondary Organic Aerosols: Browning Effects and Alterations in Oxidative Reactivity

**DOI:** 10.1021/acs.est.4c02316

**Published:** 2024-06-12

**Authors:** Liyuan Zhou, Zhancong Liang, Yiming Qin, Chak K. Chan

**Affiliations:** † Division of Physical Sciences and Engineering, King Abdullah University of Science and Technology, Thuwal, Jeddah 23955-6900, Kingdom of Saudi Arabia; ‡ School of Energy and Environment, City University of Hong Kong, Tat Chee Avenue, Kowloon 999077, Hong Kong SAR, China

**Keywords:** volatile chemical products, aerosol evaporation, brown carbon, photosensitization, reactive oxygen species

## Abstract

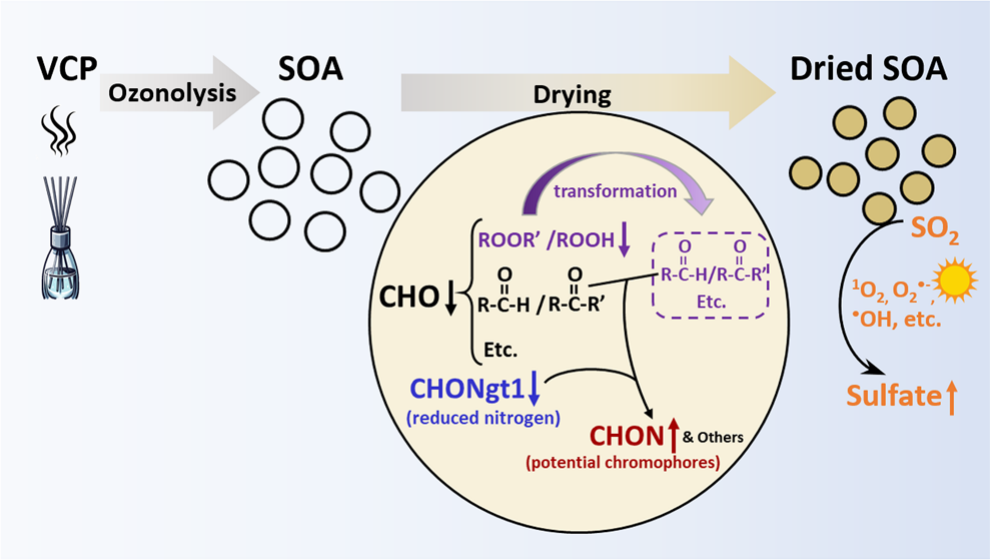

Volatile chemical products (VCPs) are increasingly recognized as significant sources of volatile organic compounds (VOCs) in urban atmospheres, potentially serving as key precursors for secondary organic aerosol (SOA) formation. This study investigates the formation and physicochemical transformations of VCP-derived SOA, produced through ozonolysis of VOCs evaporated from a representative room deodorant air freshener, focusing on the effects of aerosol evaporation on its molecular composition, light absorption properties, and reactive oxygen species (ROS) generation. Following aerosol evaporation, solutes become concentrated, accelerating reactions within the aerosol matrix that lead to a 42% reduction in peroxide content and noticeable browning of the SOA. This process occurs most effectively at moderate relative humidity (∼40%), reaching a maximum solute concentration before aerosol solidification. Molecular characterization reveals that evaporating VCP-derived SOA produces highly conjugated nitrogen-containing products from interactions between existing or transformed carbonyl compounds and reduced nitrogen species, likely acting as chromophores responsible for the observed brownish coloration. Additionally, the reactivity of VCP-derived SOA was elucidated through heterogeneous oxidation of sulfur dioxide (SO_2_), which revealed enhanced photosensitized sulfate production upon drying. Direct measurements of ROS, including singlet oxygen (^1^O_2_), superoxide (O_2_^•–^), and hydroxyl radicals (^•^OH), showed higher abundances in dried versus undried SOA samples under light exposure. Our findings underscore that drying significantly alters the physicochemical properties of VCP-derived SOA, impacting their roles in atmospheric chemistry and radiative balance.

## Abstract 2

Aerosol evaporation substantially modifies the composition of VCP-derived SOA, leading to the formation of brown carbon and enhancing oxidative reactivity under light conditions.

## Introduction

Volatile chemical products (VCPs), such as household and personal care products, cleaning agents, coatings, adhesives, and pesticides, are recognized for contributing to indoor pollutants and potential adverse health effects.^1,2^ Recent studies have extended beyond indoor air chemistry of VCP emissions, focusing on their impact on outdoor volatile organic compound (VOC) levels. In densely populated urban areas like Los Angeles and New York City, VCP emissions have become a significant source of VOCs, rivaling those from vehicular sources.^3−5^ This shift has significant implications for urban air quality as the chemical composition and behavior of VCP emissions can differ markedly from those of traditional VOC sources.

Fragranced chemical products are a significant subcategory of VCPs, which describes scented or perfumed air fresheners, cleaning agents, etc.^6^ These products consist of active ingredients and fragrance mixtures diluted into solvents or plasticizers.^7^ However, the detailed composition of these products is often obscured due to incomplete disclosure requirements (components or mixing ratios) for manufacturers.^6^ Widely used in both personal and institutional settings, air fresheners alone represent a global market exceeding US $10 billion, with usage increasing by about 8.8% annually in Korea^8^ and nearly 75% of households in the US.^9^ Understanding the atmospheric impact of these fragranced products is crucial for a comprehensive evaluation of their roles in urban air quality.

Once released into the atmosphere, emissions from VCPs could undergo atmospheric oxidation processes and contribute to the formation of secondary organic aerosol (SOA).^3,10^ Qin et al.^10^ utilized a bulk SOA yield to model SOA formation from VCP emissions and reported that VCPs contribute approximately 41% to the total anthropogenic SOA in Los Angeles during summer. Seltzer et al.^11^ integrated a comprehensive US nationwide VCP inventory into an air quality model, revealing an increase of 15–30% in the population-weighted annual average SOA in Southern California and New York City, attributable to VCP emissions. Pennington et al.^12^ indicated that VCP usage could be responsible for nearly 50% of midday anthropogenic SOA. In addition to these modeling studies, recent experimental research has focused on the SOA formation potential of individual VCP tracer compounds.^13−17^ Sasidharan et al.^18^ reported that hydrocarbons, including terpenes, branched alkanes, and cyclic alkanes, account for 60–75% of the SOA arising from VCP use, while oxygenated VOCs, such as terpenoids, glycols, and esters, constitute the rest. Despite these advancements, there remains a notable gap in understanding the characteristics of SOA formed from actual VCPs, which are expected to exhibit significantly more complex compositions. Furthermore, the formed SOA undergoes further atmospheric processing, such as a series of evaporation/dissolution cycles (cloud cycling) during their several-day lifetime,^19^ which play a crucial role in the atmospheric dynamics of these SOAs.^20,21^ The evaporation of SOA in response to decreased relative humidity leads to the loss of their volatile components and a concurrent concentration of nonvolatile fractions. This phenomenon plays a key role in accelerating condensed-phase chemistry.^22−24^ For example, formation rates of light-absorbing species dramatically increase in evaporating droplets that contain reduced nitrogen species and carbonyls (i.e., occurring on the order of seconds or less) under low RH conditions (e.g., less than 30%).^22,25,26^ A critical knowledge gap exists in understanding the chemical transformations that occur during the evaporation of SOA, particularly those derived from VCPs. There is an urgent need for more comprehensive studies on the atmospheric processing of VCP-derived SOA, especially considering the increasing prevalence of VCPs and their role in urban air pollution.

In this study, we investigate the transformation of SOA derived from a commonly used room deodorant air freshener, focusing particularly on the changes occurring during evaporation. We explore the impact of aerosol evaporation on molecular composition, mass absorption coefficient (MAC), and generation of reactive oxygen species (ROS) in VCP-derived SOA formed via ozonolysis. The selection of ozonolysis for this study is predicated on the abundant presence of terpenes in air fresheners,^6,27,28^ known for their rapid reaction with ozone,^29^ thereby providing a representative model for examining the chemical transformations in SOA. Employing advanced analytical techniques, we seek to elucidate the chemical transformations occurring during the evaporation of VCP-derived SOA samples. Additionally, we aim to compare the oxidative reactivity and photochemical reactions in both undried and dried VCP-derived SOA samples.

## Materials and Methods

Experiments were conducted using an oxidation flow reactor (OFR) to examine the chemical composition and particle size distribution of VCP-derived SOA and to collect particles onto filters for subsequent offline chemical analysis. The VCP employed in this study was a room deodorant air freshener, procured from a local supermarket and manufactured by the Kobayashi Pharmaceutical Company. This air freshener utilizes a wide filter paper to draw the scented liquid by capillary force, allowing the fragrance strength to be adjusted. It features a combined scent of apple and sweet floral notes. The disclosed ingredients of the air freshener include amino acid-based deodorizer, fragrance, and surfactant, although a comprehensive list of components was not provided. Kobayashi has a notable presence of air fresheners in the household product market,^30,31^ and it is considered a representative VCP in indoor environments.

### OFR Experiments and Sample Collection

SOA formation from gas-phase emissions of the VCP was investigated in an OFR. Approximately 250 mL of scented liquid from the air freshener was drained and maintained at ambient temperature (∼25 °C) in a 500 mL flask submerged in a water bath. The carrier gas flow rate was maintained at approximately ∼0.3 L min^–1^. Prior to entering the OFR, potential particulate matter from VCP emissions was removed by using a Teflon filter. The gas-phase VCP emissions were first diluted by purified compressed air by a factor of approximately 16, and then ∼0.3 L min^–1^ of the diluted flow was introduced to the OFR resulting in a total dilution factor of around 180. Ozone was generated by a commercial generator (Model 610, Jelight Inc., USA) with pure oxygen supplied and introduced into the OFR. The OFR, with a volume of 7.2 L and dimensions of 1 m in length and 9.6 cm in diameter, was set to a total flow rate of ∼3 L min^–1^, yielding a residence time of ∼150 s. Ozone concentration was maintained in excess at approximately 10 ppm to ensure complete consumption of precursor gases. The corresponding ozone exposure in this setup was 417 ppbv h, which is equivalent to an atmospheric aging of 8.3 h at 50 ppbv ozone concentration. The relative humidity (RH) of the outflow of OFR was monitored (M170, Vaisala Inc., Finland) and stabilized at ∼80%. Post-OFR, the SOA passed through an activated carbon denuder for gas-phase species removal and a diffusion dryer before measurement by a Scanning Mobility Particle Sizer (SMPS, model 3938, TSI, Inc.) and a Time-of-Flight Aerosol Chemical Speciation Monitor (ToF-ACSM) equipped with a PM_2.5_ lens and a capture vaporizer (Aerodyne Research, Inc.). In addition, SOA was collected on 47 mm quartz filters for approximately 15 h. These filters were prebaked at 550 °C for 12 h in a muffle furnace to remove residual organics. Two types of SOA were collected: one directly downstream of the activated carbon denuder (referred to as “undried SOA”) and the other post the activated carbon denuder and diffusion dryer (referred to as “dried SOA”) with the dried aerosol stream at ∼12–20% RH. Additionally, to investigate the effect of RH, we conducted drying experiments at higher RH levels utilizing the diffusion dryer. The aerosol drying process allows for the qualitative simulation of the evolution of VCP-derived SOA upon water and volatile component evaporation,^22^ as well as potential condensed phase reactions in the SOA. Notably, research suggests that reactions in drying aerosols occur orders of magnitude faster than in bulk aqueous solution experiments without evaporation.^22,32^ Gen et al.^33^ have reported that the brown carbon formation rate in aerosols containing glyoxal and ammonium salts rises significantly with decreasing RH or aerosol liquid water content. In the current study, employing a dryer allows for potentially accelerated reactions in the dried SOA, offering insights into the rapid chemical transformations that occur under such conditions. The quartz filter collection method introduces intrinsic positive and negative artifacts due to volatile species;^34,35^ however, the use of an activated carbon denuder minimizes potential absorption of organic gases on the filter, thereby reducing positive artifacts. Evaporation of semivolatile compounds from the filter may create negative artifacts, potentially leading to underestimation of these compounds. However, as this study does not primarily focus on the quantification of these compounds, a detailed analysis of artifact interference is not included in the manuscript.

### Filter-Based SO_2_ Uptake Experiments

Immediately after collection, the filter samples were placed in a flow cell and subjected to a regulated airflow of 1 L min^–1^, consisting of a blend of humidified and dry purified compressed air achieving 80% RH, infused with approximately 2 ppm SO_2_. This configuration facilitated the reaction of SO_2_ with SOA under conditions of either UVA irradiation or darkness. The UVA lamps provided a total irradiance of approximately 5.1 × 10^15^ photon cm^–2^ s^–1^. The specifics of the measurement are detailed in the Supporting Information (SI). Following reaction times of 2, 5, and 10 h, the sample filters were extracted under sonication in an ice bath in either acetonitrile or Milli-Q water (18.2 MΩ) for 30 min, and subsequently, the mixture was filtered through a 0.22 μm poly(tetrafluoroethylene) syringe filter. The aqueous extracts were then subjected to ion chromatography (Dionex ICS 1100, CA) measurements, specifically targeting the analysis of inorganic ions, such as sulfate and nitrate. Acetonitrile was selected over methanol to extract organic species to minimize solvent related artifacts due to the methanolysis of conjugated carbonyl compounds.^36^ The peroxide content in SOA was quantified via an iodometric–spectrophotometric method, adapted from Docherty et al.^37^ Briefly, the iodide ion (I^–^) can be oxidized to I_2_ by peroxide moieties (including H_2_O_2_, ROOH, and ROOR) under acidic conditions. I_2_ then complexes with I^–^ to form I_3_^–^, which exhibits a distinctive orange-brown coloration and strongly absorbs light at 470 nm. For the assay, the acetonitrile-extracted SOA sample was placed in a 96-well UV plate (Thermo Fisher), mixed with formic acid and potassium iodide solutions to initiate the reaction, and sealed to preclude ambient oxygen exposure. After 1 h of incubation at room temperature, the absorbance of the solution was measured at 470 nm using an absorbance microplate reader (SpectraMax M2e). The absorbance readings, calibrated against benzoyl peroxide standards, were converted to mass fractions, assuming a molecular weight of 242.23 g mol^–1^, in line with the procedure described by Ye et al.^38^

### The Molecular Composition of Gas and Particle Phase Species from the VCP

The gas phase VCP emissions were sampled in a 5 L Tedlar bag and subsequently analyzed using comprehensive two-dimensional gas chromatography–time-of-flight mass spectrometry (GC × GC-TOFMS). The GC × GC-TOFMS system comprised a gas chromatograph (8890, Agilent, California, PA, USA) and a time-of-flight mass spectrometer (7250A, Agilent, California, PA, USA) and was equipped with a one-dimensional column (DB-5MS, 0.25 mm × 30 mm× 0.25 mm, J&W Scientific, USA) and a two-dimensional column (DB-17MS, 2 μm × 0.18 μm × 0.18 μm, J&W Scientific, USA). Detailed specifications and methodological information can be found elsewhere.^39^

The acetonitrile-extracted SOA was analyzed using Fourier-transform ion cyclotron resonance mass spectrometry (FT-ICR MS) (Bruker Daltonik GmbH, Bremen, Germany) equipped with a 9.4 T refrigerated superconducting cryomagnet and ParaCell analyzer cell. Samples are ionized in positive ion mode using an electrospray ionization (ESI) ion source.^40,41^ The data was recorded with the *m*/*z* range of 100–1200, with a time-domain size of 8 mega-points. The mass spectra were first externally calibrated with sodium trifluoroacetate (NaTFA) clusters. After data acquisition, the raw data were further internally recalibrated with a known homologous series using quadratic calibration. Internal mass calibration and mass list exportation were performed using DataAnalysis version 5.2 (Bruker Daltonics GmbH & Co. KG, Bremen, Germany). Chemical formula assignments and relative abundances of compound classes were determined via Composer version 1.2 software (Sierra Analytics, Pasadena, CA, USA). The elemental composition of each monoisotopic mass peak was calculated based on the determined accurate mass, considering a heteroatom range of C_1–50_, H_1–200_, N_0–3_, S_0–3_, O_0–20_ within an error of 0.5 ppm. Blank filters were processed and analyzed by following the same procedure to exclude possible contamination. The resulting chemical formulas and their distributions were further visualized based on their double bond equivalent (DBE), carbon number, and signal contributions. For the chemical formula C*c*H*h*O*o*N*n*S*s*, the DBE was calculated using the equation: DBE = (2*c* + (2 – *h*) + *n*)/2.^41,42^

### Mass Absorption Coefficients (MAC) of VCP-Derived SOA

The absorbance of the SOA extracts was obtained by using UV–visible (UV–vis) spectroscopy (UV-3600, Shimadzu Corp., Japan). The absorption spectra obtained were presented in the form of wavelength dependent MAC, calculated according to Lee et al.^41^ using the equation: MAC(λ) = *A*_10_^solution^(λ) × ln (10)/(*b* × *C*_mass_), where *A*_10_^solution^(λ) is the base-10 absorbance at wavelength (λ) of SOA extracts in solution with known mass concentration *C*_mass_ (g cm^–3^) and *b* is path length (cm).

### Reactive Oxygen Species (ROS) Capture Experiments

ROS were identified using continuous-wave electron paramagnetic resonance spectroscopy (CW-EPR, Bruker, Germany), employing a spin-trapping technique.^43,44^ An aqueous solution of ∼100 mM 5,5-dimethyl-1-pyroline *N*-oxide (DMPO) (EnzoLife Sciences, ≥99%) served as the trapping reagent for hydroxyl radicals (^•^OH), and a methanolic solution was used for superoxide radicals (O_2_^•–^). Additionally, an aqueous solution of ∼100 mM 2,2,6,6-tetramethylpiperidine (TEMP) (Thermo Fisher, ≥98%) was employed as the trapping reagent for singlet oxygen (^1^O_2_).^45−47^ The trapping reagent solutions were uniformly dripped onto the filter punches in the quartz vial.^47^ A xenon lamp with a long-pass filter (>300 nm) was used to illuminate the suspension from a top-down angle for 30 min, and aliquots were sampled for EPR analysis immediately. Background signals due to the degradation of traps were subtracted. Spectra were recorded with a center field of 3350 G, a sweep width of 200 G, a receiver gain of 30 dB, a modulation amplitude of 1.0 G, an attenuation of 12 dB, a microwave power of 12.6 mW, a microwave frequency of 9.84 GHz, a modulation frequency of 100 kHz, a conversion time of 5.12 ms, and a time constant of 0.01 ms.

## Results and Discussion

### Chemical and Optical Properties of VCP-Derived SOA

The offline GC × GC-TOFMS analysis revealed a pronounced dominance of ester-based VOCs from VCP emissions (see Figure S2 in the SI). Esters constitute seven of the ten most abundant VOCs identified, corroborating their prevalent application in the flavor and fragrance sectors.^48^ Notably, isoamyl acetate, ethyl acetate, and ethyl 2-methylbutyrate were predominant, accounting for 36.2% of the total GC × GC-TOFMS signal, consistent with their established presence in scented air fresheners^49−51^ and a spectrum of consumer products including shampoos, conditioners, and cleaning products.^52^ Terpenes emerged as the second major group in the VCP-derived VOC profile with limonene being the most substantial, comprising 18.5% of the GC signal, followed by β-pinene (4.8%). These compounds are known for their rapid reaction with ozone to form SOA.^18,29,50^ Recent literature highlights the contribution of VCP-derived oxygenated VOCs, including esters, glycols, oxygenated aromatics, and amines, reacting with ^•^OH in SOA formation.^18^ These compounds may play a minor role in ozonolysis reactions. Moreover, nitrogen-containing species were identified, albeit at lower intensities. Compounds like methyl anthranilate, ethyl anthranilate, ethanolamine and trimethylpyrazine, each representing less than 0.3% of the total GC × GC-TOFMS signal, are notable for their scents and extensive use as fragrance components.^53^ A comprehensive list of identified species is available in the SI (Table S1).

The secondary particles resulting from the ozonolysis of VCP-derived VOCs displayed a monomodal number distribution, peaking at approximately 80 nm (refer to Figure S3). The ToF-ACSM recorded an average mass concentration of these particles at around 950 μg m^–3^, with organic matter and nitrate nitrogen (N_NO3_) constituting 97% and 3% of total nonrefractory PM_2.5_, respectively. While N_NO3_ is typically indicative of inorganic nitrate, recent findings suggest organic nitrogen species, such as nitro-compounds, N-heterocycles, and amide, can substantially contribute to NOx^+^ ion fragments.^54^ Furthermore, the signal of N_NO3_ at nominal *m*/*z* 46 and 30 in the unit resolution mass spectra may exhibit significant interference,^55^ including potential overlap between NO_2_^+^ and CH_4_NO^+^ as well as NO^+^ and CH_4_N^+^.^56^ CH_4_NO^+^ and CH_4_N^+^ have been identified in compounds that contain amine groups.^54^ Indeed, our current study found no NO_3_^–^ through IC analysis, hinting at the presence of organic nitrogen species in the SOA. The total aerosol mass concentration, measured by ToF-ACSM and calculated from SMPS measurements, exhibited a strong correlation. The estimated density of VCP-derived SOA in this study was 1.18 g cm^–3^ (*R*^2^ = 0.98, as shown in Figure S3). The average fractions of the total organic signal at *m*/*z* 43 (*f*_43_, indicative of less oxidized organic aerosols) and *m*/*z* 44 (*f*_44_, suggesting highly oxygenated organic aerosols^57^) in VCP-derived SOA, as measured by ToF-ACSM, were 0.17 and 0.07, respectively (Figure S4). These values align with the range observed in chamber studies for SOA produced from the oxidation of α-pinene or Δ-carene by ozone or NO_3_ radicals, typically 0.02–0.13 for *f*_43_ and 0.01–0.18 for *f*_44_.^58^ Moreover, the corresponding mass spectrum showed a moderate correlation (*R*^2^ = 0.73) with less-oxidized oxygenated organic aerosol factors measured around the Seoul Metropolitan Area, employing an ACSM with a capture vaporizer.^59^

**Figure 1:**
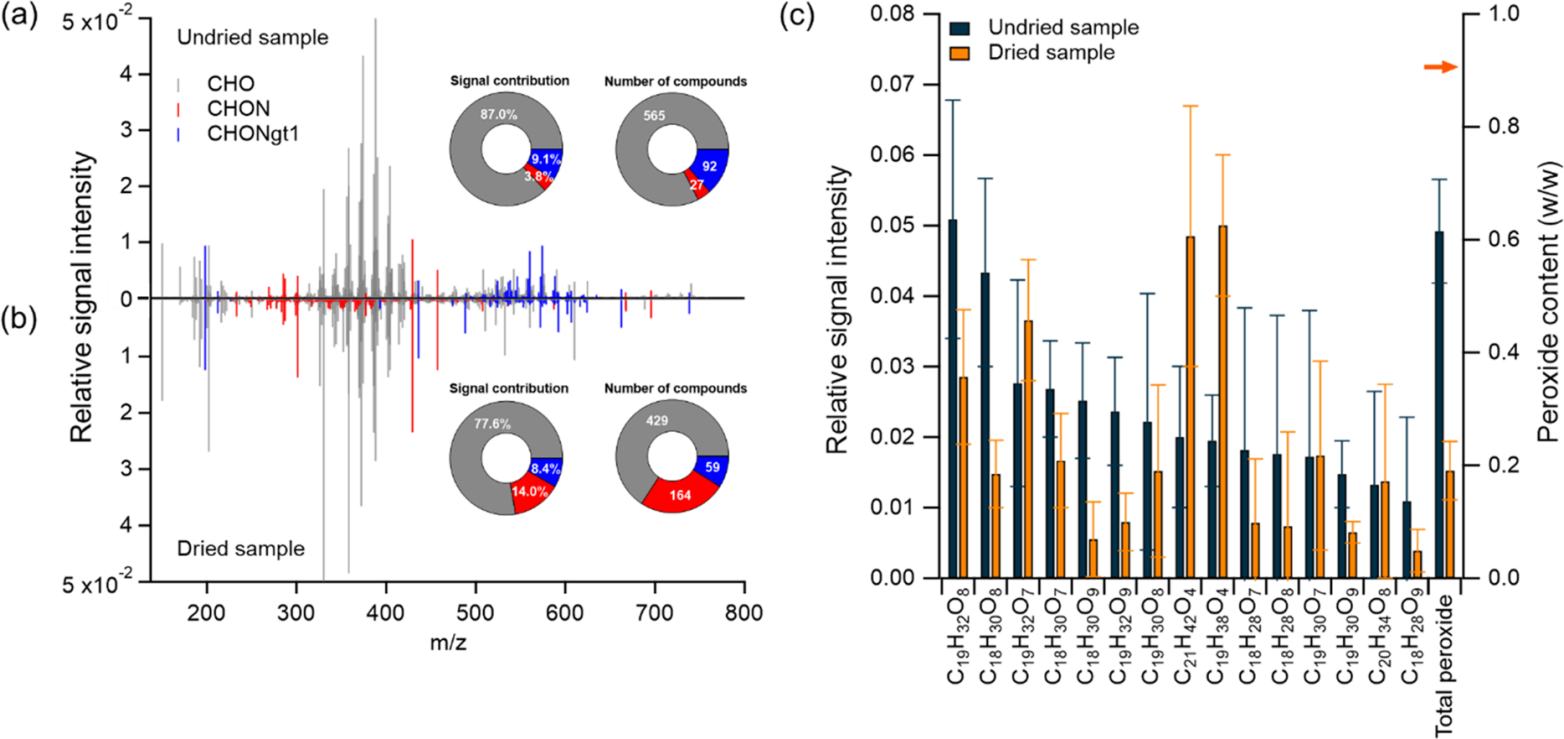
Assigned peaks in FTICR mass spectra along with signal contributions and numbers of various compound types in (a) undried and (b) dried VCP-derived SOA samples and (c) the changes of relative signal intensity of top 15 species and the total peroxide content following SOA evaporation. Error bars represent the standard deviation (1σ).

To elucidate the molecular characteristics of SOA derived from VCP, we further analyzed the samples with FT-ICR MS. Figure 1 presents the mass spectrum of acetonitrile-extracted VCP-derived SOA, analyzed in positive ESI ion mode. Compounds containing only carbon, hydrogen, and oxygen (CHO) emerged as the predominant constituents, comprising 87% of the total detectable signals (Figure 1a). The CHO mass spectral profile exhibited a multipeak distribution, akin to the pattern observed in limonene/O_3_ SOA, spanning monomeric to trimeric SOA constituents. Conforming to the structural classifications of limonene/O_3_ SOA by Bateman et al.,^60^ the mass spectrum displayed distinct clusters within monomeric (<300 *m*/*z*), dimeric (300–500 *m*/*z*), and trimeric (500–700 *m*/*z*) ranges, corresponding to one, two, and three oxygenated limonene units, respectively. The dimeric range, particularly, accounted for the majority of signals (65%), suggesting the formation of dimers through alkylperoxy radical (RO_2_^•^) reactions. The top five signal contributors were C_19_H_32_O_8_, C_18_H_30_O_8_, C_19_H_32_O_7_, C_18_H_30_O_7_, and C_18_H_30_O_9_, which were also reported in limonene/O_3_ SOA studies^61−63^ and likely represent ROOR-type peroxides (Figure 1a). We also observed a range of alcohols, carbonyls, and peroxide within the monomeric spectrum, such as C_10_H_18_O_4_, C_9_H_14_O_4_, C_9_H_14_O_3_, and C_9_H_16_O_4_, potentially resulting from varied radical reactions during limonene ozonolysis.^62^

The drying of the aerosols, as shown in Figure 1b, has only a minor effect on the distribution of the CHO compounds. Figure 1c shows a comparative analysis of the top 15 species in undried VCP-derived SOA and their respective relative signal intensities in the dried samples. Thirteen out of the top 15 species were potential ROOR-type peroxides derived from limonene/O_3_ SOA, among which 10 species exhibited a substantial reduction in relative signal intensity of 31–78% in dried particles. This is in line with the decreased total peroxide fractional content quantified via iodometric-spectrophotometric methods that decreased from 0.61 to 0.19 on average after aerosol evaporation. The decay of peroxides can be attributed to nonreactive loss (i.e., evaporation) and chemical reactions.^64^ The relative signal intensity-weighted average saturation mass concentration (C*) and thus the volatility of the CHO compounds were calculated following the approach of Li et al.^65^ The C* values for CHO compounds in undried and dried SOA were 8.9 × 10^–4^ and 5.6 × 10^–3^ μg m^–3^, respectively, placing them within the low-volatile organic compounds (LVOC) range of 3 × 10^–4^ < C* < 0.3 μg m^–3^.^66^ Consequently, evaporation is deemed to have a negligible role in this study. Conversely, the evaporation of water elevated the concentrations of remaining solutes, such as low-volatile peroxides and other reactants, which accelerates their reactions.^22^ At high concentrations, organic peroxides are more prone to undergo unimolecular or bimolecular reactions with other organic compounds, leading to the formation of carbonyls, carboxylic acids, esters or alcohols.^67,68^ Among the top 15 identified species, two molecules, C_21_H_42_O_4_ and C_19_H_38_O_4_, exhibit molecular formulas that are too saturated to be direct products of limonene oxidation. They exhibited an increased relative signal intensity postevaporation (Figure 1c), suggesting formation via alternative reaction pathways within the complex organic matrix. Furthermore, nitrogen-containing compounds with one or more nitrogen atoms (CHON and CHONgt1) were detected, likely originating from the oxidation of nitrogenous precursors. This may occur either through ozonolysis of unsaturated carbon–carbon bonds in their backbones or of nitrogen-containing functional groups.^69,70^ They accounted for 3.8% and 9.1% of the total signals of compounds detected in undried SOA, respectively (Figure 1a). Aerosol evaporation induced a slight decline in the relative signal contribution of CHONgt1 compounds of 0.7% but a pronounced decrease of nearly 10% in CHO species, while CHON species exhibited an approximate 10% increase (Figure 1b). In terms of the number of detected species, 684 species were observed in the undried VCP-derived SOA samples (only including peaks that appeared in at least three of the mass spectra of different filter samples) (Figure 1a). Of those, 220 species were no longer detectable post evaporation, and they were mainly CHO and CHONgt1 species. In the dried samples, 652 species reproducibly appeared. Of those, 188 species correspond to newly formed, and 464 species correspond to those present in the undried sample. Among newly formed species in dried samples, 87% of them are CHON compounds (Figure 1b).

**Figure 2:**
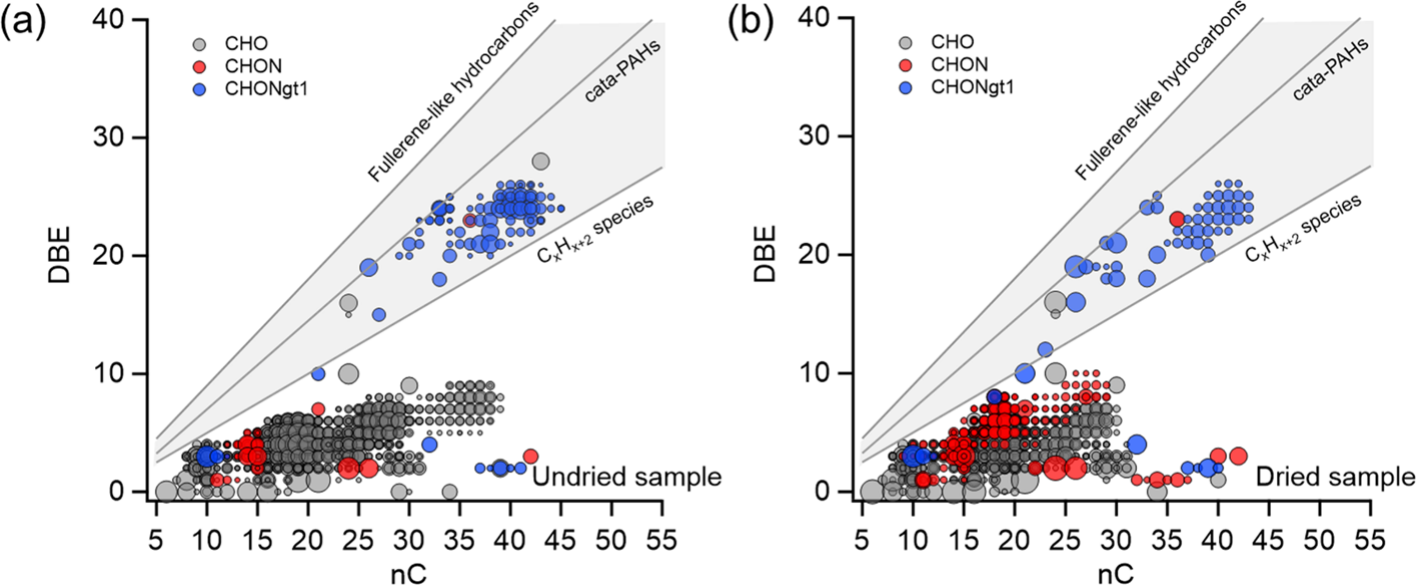
Plots of DBE vs number of carbon atoms derived from assigned mass spectra of undried and dried VCP-derived SOA. Symbol size is proportional to the logarithmic relative intensity of corresponding peaks. Lines represent reference DBE values of C_*x*_H_*x*+2_ species, *cata*-condensed PAHs, and fullerene-like hydrocarbons.

**Figure 3:**
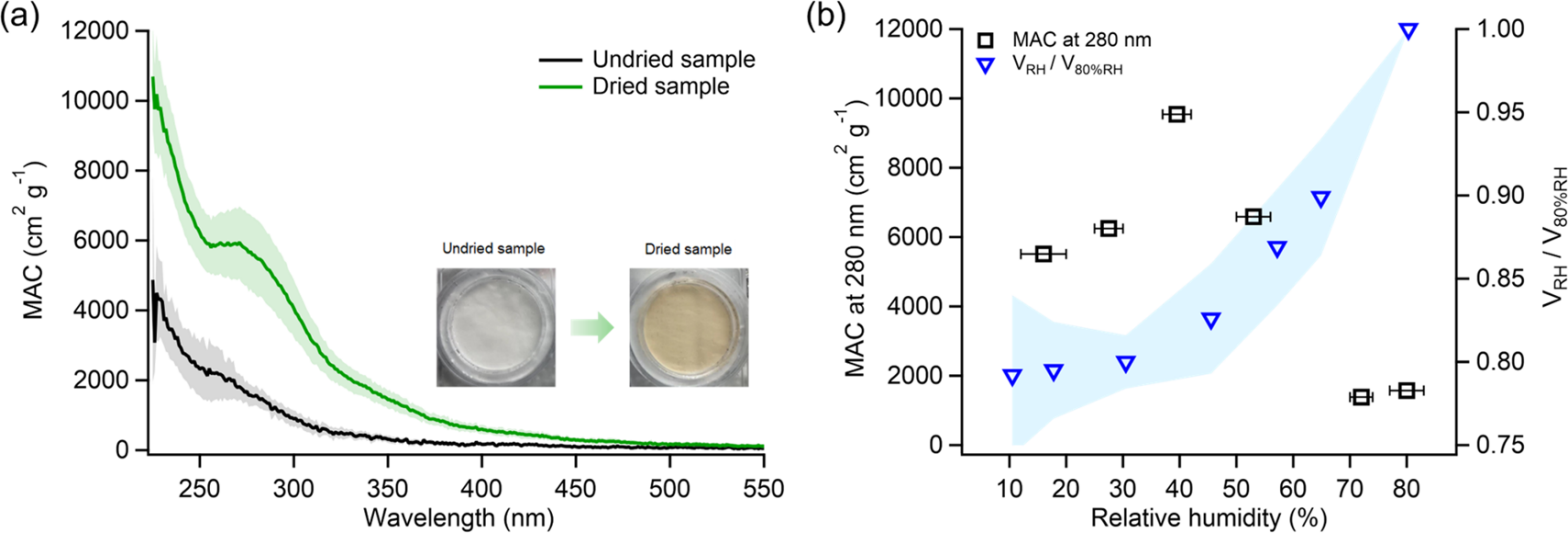
(a) UV–vis absorption spectra for both undried and dried VCP-derived SOA. (b) Variations in the volume ratio of VCP-derived SOA at specific RHs relative to 80% RH, alongside the mass absorption coefficient (MAC) at 280 nm across different RH levels. Shaded areas represent the standard deviation (1σ).

Figure 2 plots the DBE, the total number of double bonds and rings calculated on the basis of assigned formulas, of VCP-derived SOA, as a function of the number of carbon atoms in their structures.^40,71^ The data are presented alongside reference DBE values characteristic of the highest possible number of conjugated double bonds in linear polyenes with a general formula C_*x*_H_*x*+2_ and the highest possible number of double bonds and cycles in cata-condensed PAHs and fullerene-like hydrocarbons.^72^ The CHONgt1 compounds possess the highest DBE values and carbon number, indicative of their conjugation and potential contribution to the mild absorption of UV–vis light by the undried VCP-derived SOA (Figure 3a). Figure 3a compares UV–vis absorption spectra of undried and dried VCP-derived SOA samples. Following evaporation, the MAC values went up by a factor of 4.1 and 3.7 at 280 and 400 nm, respectively, in the dried VCP-derived SOA, resulting in a discernible browning of the filter samples. Additionally, the absorption spectra of dried VCP-derived SOA samples, extracted immediately and 2 days after collection (stored at low RH condition), were analyzed (refer to Figure S5). The similarity in absorption intensity between these two cases, without additional formation of light-absorbing species over a two-day interval on the filter, indicates that such species predominantly form in aerosols during evaporation, rather than on the filter postcollection. This suggests that aerosol liquid water plays a crucial role in generating light-absorbing materials.^22^ Consequently, it appears the VCP-derived SOA transformation predominantly occurs at moderate RH levels during the RH reduction from 80%, rather than at low RHs (12–20%). The residence time of aerosol particles in the diffusion dryer is approximately 5s, representing an upper limit for the evaporation time scale;^22^ it is likely that the browning reaction takes place on a time scale of seconds or less. On the other hand, for the undried sample, the possibility of further reactions taking place on the filter under wet conditions cannot be ruled out, potentially contributing to its mild UV–vis light absorption. Table 1 lists the average elemental compositions of undried and dried VCP-derived SOA samples. The O/N of 0.971 of CHONgt1 species indicates the presence of reduced nitrogen groups. Typically, compounds with an O/N ratio below 3 are likely to contain a reduced nitrogen group alongside other oxygenated functional groups.^73−75^ In the undried samples, CHON species were limited in number, primarily found in the carbon number range 11–15 and with a DBE of less than 5 (Figure 2a). However, in the dried SOA samples, there was a notable formation of abundant CHON compounds. These newly formed CHON compounds, proximal to the C_*x*_H_*x*+2_ limit, such as C_26_H_35_NO_7_, C_27_H_37_NO_7_, C_28_H_39_NO_7_, and C_27_H_39_NO_7_ with DBE values of around 10, potentially feature up to 10 conjugated bonds. They emerge as plausible chromophores for brown carbon, particularly when nitrogen atom positioning favors charge transfer mechanisms similar to the classic case of cyanine dyes.^40^ Comparing their DBE values with the C_*x*_H_*x*+2_ reference indicates that π-bond conjugation in these molecules extends across a significant portion of their carbon framework, though not entirely. The presence of cyclic structures could further reduce the length of the conjugated segments.^40^ In addition to a notable decrease in the signal contribution and the number of detected compounds for CHO species, several CHONgt1 species, such as C_39_H_34_N_2_O, C_40_H_36_N_2_O, C_33_H_24_N_4_O_4_, and C_30_H_22_N_2_O_10_ also diminished significantly upon aerosol evaporation. Most of these species, likely containing reduced nitrogen groups as indicated by their O/N ratios significantly lower than 3, exhibited a reduction exceeding 85% postevaporation. Reactions between reduced nitrogen species, such as organic amines and imines, amino acids, ammonia and ammonium, and carbonyl groups can produce imines or N-heterocyclic compounds, depending on the parent carbonyl structures.^32,76,77^ Updyke et al.^76^ reported that the reactions of SOA compounds with ammonia resulted in the production of light-absorbing brown carbon compounds, with the extent of browning ranging from no observable change for isoprene SOA to visible change in color for limonene SOA, likely through the conversion of carbonyls to imines.^40^ Our absorption spectrum lacked the characteristic peak at 500 nm observed in limonene/O_3_ SOA aged with ammonia,^41,76^ suggesting alternative chromophore formation in our study (Figure 3a). The observed contrasting trends in the signal contributions and the number of detected compounds for CHO and CHON upon evaporation, as shown in Figure 1a,b, suggest a potential transformation of CHO to CHON species. This likely takes place in the condensed phase, as the removal of gas-phase species is achieved by using the activated carbon denuder. During this process, pre-existing carbonyls in the SOA become more concentrated due to water evaporation. Furthermore, additional carbonyls may be transformed from peroxides through mechanisms such as Hock rearrangement.^78^ We postulate that this dual effect, the concentration of existing carbonyls and the generation of new carbonyls from peroxides, could enhance interactions with reduced nitrogen groups present in CHONgt1 compounds. This interaction leads to the formation of compounds exhibiting higher DBE values (CHON) than parent CHO species (Figure 2b). Moreover, the potential conversion of carbonyl to imine or N-heterocyclic structures may explain the observed decline in the O/C ratios of CHON compounds relative to CHO compounds (Table 1 and Figure S6). However, CHONgt1 compounds with high DBE, being involved in the reaction, may diminish and thus potentially reduce the absorption of the SOA. The precise molecular identities of the individual chromophores in this study remain unidentified. It is possible that the chromophores, while representing only a minor portion of the signal intensities, contribute significantly to absorbance.^79^ Furthermore, Sharpless et al.^80^ reported that the absorbance of chromophoric dissolved organic matter may not be a simple sum of spectra of the individual chromophores because of chromophore–chromophore electronic interactions, e.g., charge transfer interactions. Light-absorbing compounds may arise from aldol condensation reactions involving aldehydes,^81−83^ further contributing to the complex absorption changes within the VCP-derived SOA. Overall, these findings underscore the possibility of yielding light-absorbing compounds in VCP-derived SOA from the intricate network of reactions.

**Table 1:** Average Elemental Composition, Elemental Ratios, and Double Bond Equivalents (DBE) of Undried and Dried VCP-Derived SOA

	Compd type	⟨C⟩	⟨H⟩	⟨O⟩	⟨N⟩	⟨H/C⟩	⟨O/C⟩	⟨N/C⟩	⟨O/N⟩	⟨DBE⟩
Undried VCP-derived SOA	CHO	19.4	32.7	8.0	-	1.681	0.410	-	-	4.1
	CHON	20.1	36.5	5.1	1.0	1.815	0.252	0.050	5.074	3.4
	CHONgt1	34.6	34.1	2.3	2.3	0.985	0.065	0.067	0.971	19.7
Dried VCP-derived SOA	CHO	18.3	32.0	6.9	-	1.745	0.376	-	-	3.3
	CHON	20.8	36.0	5.1	1.0	1.728	0.244	0.048	5.075	4.3
	CHONgt1	27.5	31.7	5.5	2.9	1.155	0.201	0.105	1.920	14.1

We further adjusted the diffusion dryer to varying RH levels of approximately 25–30%, 37–42%, 50–56%, and 70–74% for VCP-derived SOA drying to delve deeper into the effect of RH. The absorption characteristics of SOA conditioned at these RH levels were similar, showing a prominent peak at around 280 nm and a tail extending to 500 nm, with the MAC being highest at 37–42% RH (Figure S7). Using a hygroscopicity-tandem differential mobility analyzer (HTDMA), we examined the water content variation in VCP-derived SOA across different RHs. With decreasing RH, we observed a gradual decrease in the normalized aerosol volume (relative to that at 80% RH), which levels off at around 30% RH. This leveling off aligns with the RH where the MAC at 280 nm starts to decline (Figure 3b), indicating the onset of solidification and a possible rise in viscosity that could inhibit the formation of further light-absorbing compounds under drier conditions. The low RH conditions we employed (12–20%, referring to the “dried samples” unless stated otherwise) enabled reactions to take place solely during aerosol evaporation in the dryer, as solute concentration increased but before aerosol solidification, thereby preventing further reactions in the solidified particles on the filter and facilitating comparison with existing literature.^22^ We advocate for further mechanistic research on VCP-derived SOA browning across a range of RH levels.

### Probing the Oxidative Reactivity of VCP-Derived SOA via Sulfur Dioxide (SO_2_) Oxidation

**Figure 4:**
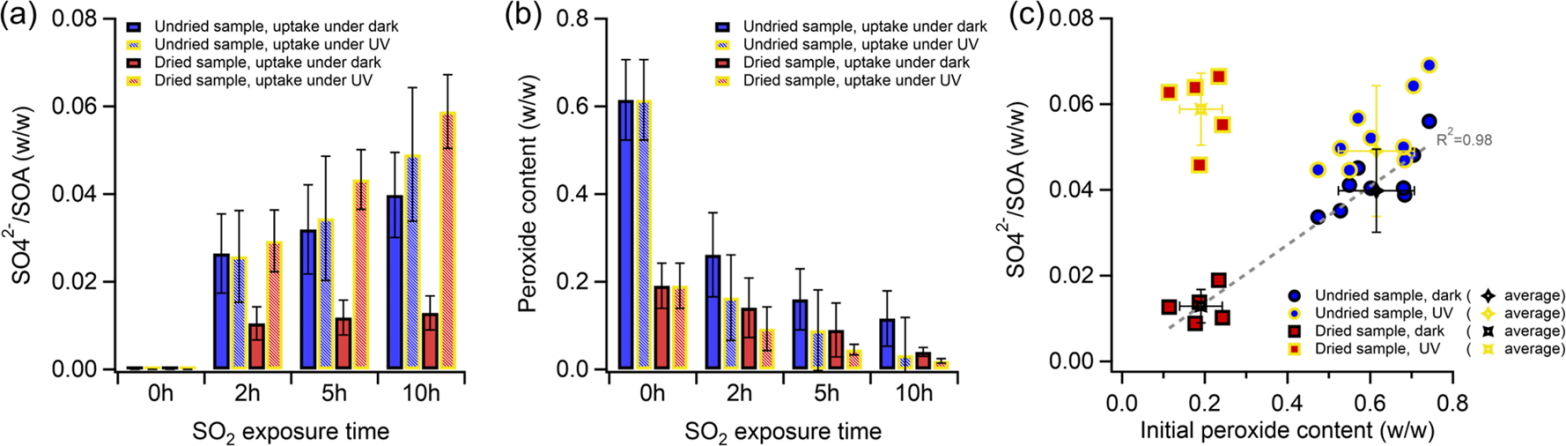
Evolution of aerosol composition upon 2, 5, and 10 h of reaction with 2 ppm of SO_2_ at 80% RH (filter-based uptake experiment): (a) Production of sulfate per mass of SOA, (b) decay of total peroxide in SOA upon SO_2_ exposure time, and (c) relationship between sulfate production upon 10 h of SO_2_ uptake and initial peroxide content in SOA. Error bars represent the standard deviation (1σ).

SO_2_, a pivotal aerosol precursor, undergoes multiphase oxidation to form sulfate, a major component of particulate matter contributing to haze events.^84^ In this context, we introduced SO_2_ to interact with VCP-derived SOA under light and dark conditions to explore the oxidative reactivity of VCP-derived SOA and its atmospheric implications. The production of sulfate and the decay of total peroxide were measured for SOA filter samples exposed to 2 ppm of SO_2_ at 80% RH. Figures 4a and 4b compare sulfate production and peroxide decay in VCP-derived SOA particles, both undried and dried, under dark and UV conditions over varying SO_2_ exposure times. In general, sulfate production in VCP-derived SOA particles increased with prolonged SO_2_ exposure under both dark and UV conditions, aligning with the observed decay of the peroxide content within these particles. The average total peroxide content in undried VCP-derived SOA particles was found to be 3-fold higher than in dried particles, as indicated in Figure 1c. Additionally, sulfate production in the undried particles was observed to be 2.5–3.1 times that of dried particles following 2–10 h of SO_2_ exposure in dark conditions. Figure 4c shows a linear dependence of sulfate production at 10 h on initial peroxide content in VCP-derived SOA particles under dark conditions, highlighting organic peroxides as potential key contributors to SO_2_ uptake and oxidation under dark. Similarly, Ye et al.^38^ reported a decline in the total particulate peroxide content concurrent with a decrease in gas-phase SO_2_ during the ozonolysis of limonene with SO_2_ under humid conditions. Recent laboratory kinetic studies have provided concrete evidence of multiphase reactions between SO_2_ and peroxides within aerosols, suggesting that aerosols rich in peroxides could be a significant contributor to sulfate formation in submicron particles.^67,85−89^

Under UV irradiation, a modest increase in sulfate production was observed in undried VCP-derived SOA particles (average increase of 19%, Student’s *t*-test, *p*-value <0.005), whereas a significant increase, reaching up to 4.6 times, was observed in dried particles after 10 h of exposure (Student’s *t*-test, *p*-value < 0.005). Notably, despite having lower initial peroxide content, the dried particles generated the highest sulfate levels under UV conditions, indicating that factors other than peroxides might be enhancing sulfate production. Recent studies have increasingly focused on the multiphase photochemical oxidation of SO_2_,^90^ involving particulate nitrate photolysis,^91,92^ photosensitization chemistry,^93−97^ and photochemical reactions occurring on mineral dust surfaces.^98,99^ Given the absence of nitrate peaks in our IC analysis, nitrate photolysis is unlikely to play a significant role in our system. Similarly, the involvement of mineral dust, which facilitates electron–hole pair generation in metal oxides, is also deemed improbable due to its absence in our experimental setup. Therefore, we hypothesized that the enhanced sulfate production from dried VCP-derived SOA particles under UV irradiation was mainly due to photosensitization. Briefly, atmospheric photosensitizers absorb light and are excited into a singlet state that evolves into a long-lived triplet state (^3^C*) via intersystem crossing (ISC). ^3^C* can react with another molecule (e.g., O_2_) to form secondary oxidants such as singlet oxygen (^1^O_2_), superoxide (O_2_^•–^), hydroperoxyl radical (^•^HO_2_), and hydroxyl radicals (^•^OH).^100−102^ Because of the substantial overlap between the CHONgt1 compounds observed in the dried and undried VCP-derived SOA, it is unlikely that these compounds significantly contribute to the enhanced sulfate production observed in dried particles. On the other hand, the newly formed abundant CHON species in the dried samples, likely resulting from interactions between carbonyls and reduced nitrogen, could potentially act as photosensitizers. These species, possibly including imines or N-heterocyclic structures with C=N bonds, could facilitate ISC from π–π*/n−π* singlet to n−π*/π–π* triplet states, a transition allowed due to compensation between angular and orbital momentum. Furthermore, Tang et al.^94^ recently reported that certain CHON species in aged incense burning particles, such as nitroaromatic compounds, can potentially undergo reduction, forming nitroxyl anion radicals. These radicals, upon reaction with O_2_, produce O_2_^•–^, further facilitating SO_2_ oxidation. However, the precise structure of these photosensitizers in the current study remains unidentified.

**Figure 5:**
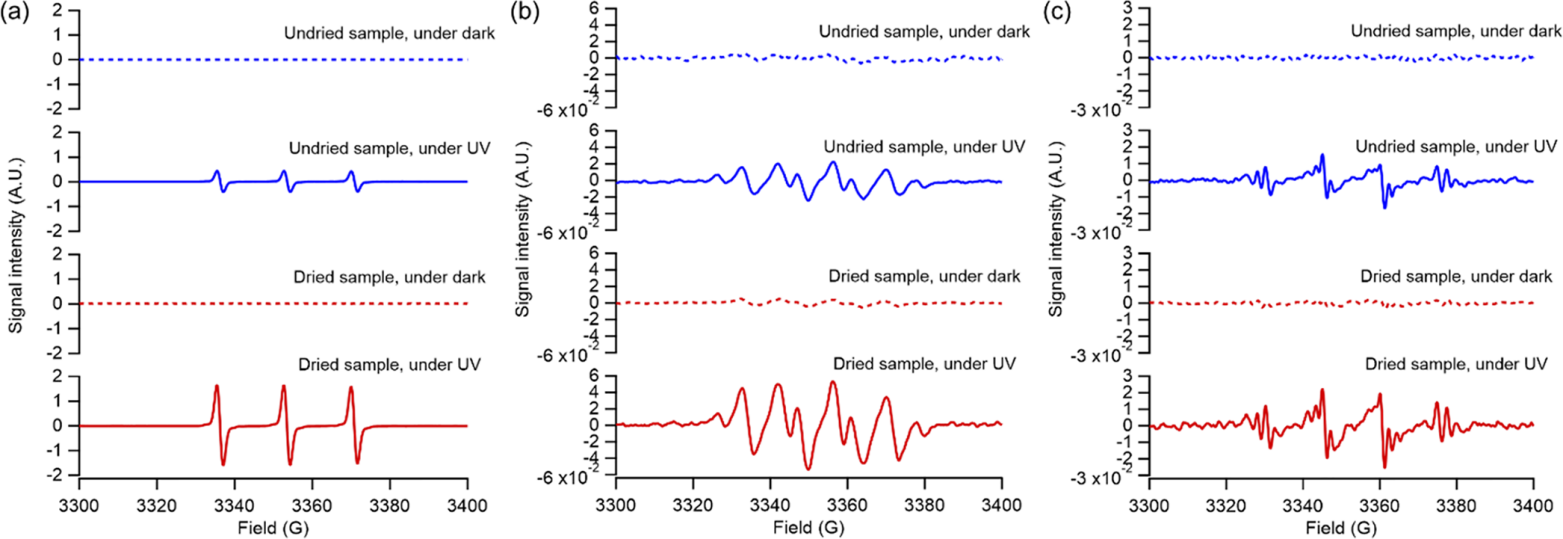
EPR spectra obtained using (a) TEMP spin trap for singlet oxygen (^1^O_2_), (b) DMPO spin trap for superoxide (O_2_^•–^), and (c) DMPO spin trap for hydroxyl radicals (^•^OH) in undried and dried VCP-derived SOA samples under dark and UV conditions. Note that superoxide was trapped in methanol, while other species were trapped in an aqueous solution. Alkoxyl radicals (^•^OR) may also be present in (c) however, their peaks were not resolved due to the low signal-to-noise ratio.

EPR spectroscopy offers insights into the potential role of photosensitization in this system. Figure 5 presents the EPR spectra for ^1^O_2_, O_2_^•–^, and ^•^OH. Notably, ^1^O_2_ signals were absent in dried VCP-derived SOA samples prior to UV exposure but showed a significant increase after irradiation. The energy to promote O_2_ to ^1^O_2_ is 94 kJ mol^–1^,^103^ lower than the reported triplet energy in typical environmental samples (∼250 kJ mol^–1^),^104,105^ suggesting that ^1^O_2_ formation through energy transfer from ^3^C* is thermodynamically favorable. Direct excitation of O_2_ to ^1^O_2_ is not feasible at actinic flux; hence, the formation of ^1^O_2_ was used as an indicator of photosensitized chemistry.^105^ The detection of O_2_^•–^ suggests potential electron transfer from triplets to dissolved oxygen, a thermodynamically favorable process given the one-electron redox potential of O_2_ (*E*°(O_2_/ O_2_^•–^) = −0.18 V) is higher than the triplet state redox potentials (*E*^o^*(^3^C*/C^•+^)) identified in environmental samples.^105^ The presence of ^•^OH in the EPR spectra could result from electron transfer from H_2_O/OH^–^ to triplets, with the high redox potential of H_2_O/OH^–^ (*E*°(OH^–^/OH^•^) = ∼2 V) allowing effective oxidation only by stronger triplet oxidants (*E*^o^*(^3^C*/C^•–^)> 2 V), explaining the low spin count of DMPO–OH (i.e., OH yield). Under UV-induced photodissociation, peroxides may yield ^•^OH and alkoxyl radicals (^•^OR);^67^ however, we did not distinguish these species due to the potential overlap in peak locations of DMPO-^•^OR and DMPO-^•^OH spin adducts. Nevertheless, the generation of ^•^OH and ^•^OR from peroxides might be of lesser significance in this study, as dried VCP-derived SOA samples, which had a lower initial peroxide content, exhibited higher signals (Figure 5c). Weak signals of O_2_^•–^ and ^•^OH were observed under dark conditions, potentially produced from the decomposition of VCP-derived SOA in solvents.^44,106,107^ These processes are significantly less efficient compared with photochemically driven reactions.

Overall, the abundances of ^1^O_2_, O_2_^•–^, and ^•^OH radicals in dried VCP-derived SOA samples were observed to exceed those in undried samples, with strong correlations identified between the differential sulfate production and the differential EPR signal peak heights under UV and dark conditions for all ^1^O_2_, O_2_^•–^, and ^•^OH radicals (*R*^2^ = 0.92–0.95) (Figure S8). This suggests the potentially important role of photosensitization in SO_2_ oxidation. However, due to the inability to directly detect ^3^C* in CW-EPR measurements, the specific contributions of photosensitized sulfate production by each radical type remain undefined in this study. Nevertheless, these results collectively point to a notable increase in the oxidative reactivity of dried VCP-derived SOA under light, contributing to both sulfate formation and potential reactive oxygen species, thereby accentuating the significance of drying processes in altering the chemical dynamics of VCP-derived SOA in the atmosphere.

## Atmospheric Implications

This study highlighted the potential for the accelerated formation of light-absorbing species in the VCP-derived SOA following aerosol evaporation. Employing a room deodorant air freshener as a model VCP, we observed enhanced absorption in dried VCP-derived SOA samples relative to their undried counterparts. While variations may exist in the constituents of air fresheners such as surfactants and fragrances, terpenes like limonene, α-pinene, and β-pinene are commonly found in a wide range of VCPs including air fresheners, shampoos, antiperspirants, body lotions, and cleaning supplies.^51,52,108^ Similarly, amines, often featured in fragrances and perfumes,^109^ and amino acid–based deodorizers are prevalent components in air fresheners and cleaning detergents,^110−112^ suggesting their widespread application across a range of VCP products. The interaction of terpenes with ambient ozone can lead to the production of carbonyls, which, in turn, can react with amine-containing compounds to form light-absorbing species. Considering the prevalent presence of terpenes and amine compounds in various VCPs, the observed reaction pathway is likely not exclusive to the air freshener used in our study. Moreover, the interaction between carbonyls and amines is not unique, as analogous reactions are noted with dissolved ammonium or ammonia.^23,40,76^ Recent studies have documented a rise in ambient ammonia concentrations across extensive regions in the U.S. and China,^113−115^ underscoring its potential significance in interacting with VCP-derived SOA. VCP-derived SOA containing carbonyls and amine-containing compounds, subjected to multiple evaporation cycles, especially through cloud cycling, can markedly enhance the formation of light-absorbing compounds. The average MAC value of the dried VCP-derived SOA was approximately 570 cm^2^ g^–1^ over the visible spectrum (300–700 nm), suggesting a modest yet noteworthy global radiative impact when compared to biomass burning aerosols, which exhibit MAC values on the order of 10^3^–10^4^ cm^2^ g^–1^.^116^ This finding underscores the potential contribution of indoor sources, specifically the complex matrix of VCPs, to atmospheric brown carbon formation upon evaporation processing. Such contributions, previously overlooked, warrant further investigation for a comprehensive understanding of their atmospheric implications. The MAC values of products from VCP-derived SOA evaporation notably increase with decreasing RH, peaking at a moderately low RH level (around 40%), and then decrease at lower RHs. This pattern is consistent with the absorption characteristics of BrC formed from reactions of glyoxal and methylglyoxal, with ammonium sulfate or glycine in droplets after drying, as reported by Kasthuriarachchi et al.^25^ These findings underscore the critical role of RH in influencing BrC formation from VCP-derived SOA. Therefore, future mechanistic research exploring the light-absorbing species originating from VCP-derived SOA under varying RH conditions is essential.

Additionally, this study sheds light on the oxidative reactivity of VCP-derived SOA, linking sulfate formation to the presence of reactive oxygen species in both undried and dried VCP-derived SOA. The ozonolysis of terpenes in VCPs leads to the formation of peroxides, contributing to 61% of the SOA mass. Upon exposure to SO_2_, a clear decay in the total peroxide content aligns with significant sulfate formation under dark conditions. The sulfate formation is further enhanced under UV irradiation, particularly in dried VCP-derived SOA particles with low initial peroxide content. The formation of new CHON species in dried VCP-derived SOA upon evaporation could potentially act as photosensitizers, leading to an increased abundance of oxidants (e.g., ^1^O_2_ and O_2_^•–^) and subsequent photosensitized sulfate formation under UV irradiation. This UV-driven sulfate formation was found to be 36% higher in partially dried samples compared to fully dried samples (Figure S9), aligning with the observation of increased BrC formation at moderate RH levels. In the broader context, recent estimates suggest that VCPs exceeding mobile sources are major contributors to fossil SOA, with consumer VCPs surpassing industrial VCPs in urban areas.^3^ Our findings add an intriguing dimension to indoor chemistry, potentially linking the evolution of VCPs and their dried counterparts to changes in the oxidative environment, thus impacting urban sulfate dynamics significantly. Furthermore, the peroxides and other ROS generated from VCP-derived SOA may contribute to health issues associated with oxidative stress in the lungs. These compounds can cause significant damage to biological tissues and cell components, potentially leading to acute airway inflammation and cardiopulmonary diseases.^44^ This underscores the need for further research into the health impacts of VCP-derived SOA. Overall, this research underscores the complex and dynamic nature of VCP-derived SOA chemistry and its implications for atmospheric science. It highlights the need for further studies to explore the interaction between indoor and outdoor aerosol chemistry and its impact on the global atmospheric system.

## Supporting Information Available

Measurement details of photon flux; chemical analysis of gas and particle species; absorption spectra for undried and dried VCP-derived SOA; correlation analysis between sulfate production and ROS generation in VCP-derived SOA samples (PDF)
