# Supplementary material for: Evaporation-Induced Transformations in Volatile Chemical Product-Derived Secondary Organic Aerosols: Browning Effects and Alterations in Oxidative Reactivity
Source: Environ Sci Technol. 2024 Jun 12;58(25):11105–17. doi: 10.1021/acs.est.4c02316 (PMC11210209; doi:10.1021/acs.est.4c02316)
Supplement: Supplementary file 1 — es4c02316_si_001.pdf [file es4c02316_si_001.pdf]

Supplemental information Zhou ***et al.***

Evaporation-Induced Transformations in Volatile Chemical Product-Derived Secondary Organic Aerosols: Browning Effects and Alterations in Oxidative Reactivity

Liyuan Zhou[1,2], Zhancong Liang[1], Yiming Qin[2] and Chak K. Chan[1*]

[1]Division of Physical Sciences and Engineering, King Abdullah University of Science and Technology, Thuwal, Jeddah 23955-6900, Kingdom of Saudi Arabia

[2] School of Energy and Environment, City University of Hong Kong, Tat Chee Avenue, Kowloon 999077, Hong Kong SAR, China

Correspondence to: Chak K. Chan (chak.chan@kaust.edu.sa)

Number of pages: 11

Number of Figures: 9

Number of Tables: 1

Description of measurement of the photon flux

Figure S1, Photon flux in the flow cell and during typical haze days or clear days in Beijing, China.

Figure S2, GC×GC chromatogram of VOC emissions from the VCP.

Figure S3, Particle number size distribution of the VCP-derived SOA and total aerosol mass from ToF-ACSM measurements vs. mass estimated from SMPS measurements.

Figure S4, Average mass spectra of the VCP-derived SOA measured by ToF-ACSM.

Figure S5, Absorption spectra of dried VCP-derived SOA filter samples extracted immediately and 2 days after the collection.

Figure S6, Van Krevelen plot of H:C vs. O:C obtained from assigned mass spectra of undried and dried VCP-derived SOA.

Figure S7, UV-Vis absorption spectra of undried and dried VCP-derived SOA and those dried at higher RH levels.

Figure S8, Relationship between the difference in sulfate production and the difference in EPR signal peak height under UV and dark conditions.

Figure S9, The normalized sulfate production ratio of UV to dark conditions for dried sample and partially dried sample.

Table S1, Chemical profile identified in VOC emissions from the VCP.

Text S1. Measurement of the photon flux.

In this study, 2-nitrobenzaldehyde (2NB), a recognized chemical actinometer, was utilized to determine the photon flux in the flow cell, in accordance with the method described by Liang et al.[1] This procedure involved the introduction of 50 μM 2NB aqueous solution into the flow cell using syringe aliquots. Subsequently, aliquots were periodically taken for photolysis monitoring. Every 5 minutes, an aliquot was collected and transferred to a sample vial, continuing for a total duration of 25 minutes, to determine the concentration of 2NB. The quantification of 2NB was performed using Ultra-High-Performance Liquid Chromatography with Photodiode Array detection (UHPLC-PDA; UHPLC, Waters Acquity H-Class, Waters, Milford, USA), with the detailed settings referenced in prior literature[2]. The UV absorption channel at 254 nm was specifically employed for the measurement of 2NB. The concentration of 2-nitrobenzaldehyde (2NB) demonstrated an exponential decay during photolysis, and the corresponding decay rate constant was determined using the following equation:

$$ln\left(\frac{[2NB]_t}{[2NB]_0}\right) = -j(\text{2NB}) \times t, \qquad (1)$$

where $[2NB]_t$ and $[2NB]_0$ are the 2NB concentrations at time t and 0, respectively. The following equation can also be used to calculate j(2NB):

$$j(\text{2NB}) = 2.303 \times (10^3\ \text{cm}^3\ \text{L}^{-1} \times 1\ \text{mol}/\text{N}_A) \times \sum (I_\lambda \times \Delta\lambda \times \varepsilon_{2NB,\lambda} \times \Phi_{2NB}), \qquad (2)$$

where N_A is Avogadro's number, I_λ is the actinic flux (photons cm^{-2} s^{-1} nm^{-1}), $\Delta\lambda$ is the wavelength interval between actinic flux data points (nm), and $\varepsilon_{2NB,\lambda}$ and Φ_{2NB} are the base-10 molar absorptivity (M^{-1} cm^{-1}) and quantum yield (molecule photon^{-1}) for 2NB, respectively. Values of $\varepsilon_{2NB,\lambda}$ at each wavelength under 298 K and a wavelength-independent Φ_{2NB} were adapted from Galbavy et al.[3]

The spectral shape of the photon output of our illumination system (i.e., the relative flux at each wavelength) was measured using a high-sensitivity spectrophotometer (Brolight Technology Co. Ltd, Hangzhou, China). Using a scaling factor (SF), this measured relative photon output, $I_{\lambda,\ relative}$, is related to I_λ as follows:

$I_\lambda = I_{\lambda,\ relative} \times \mathrm{SF}$, (3)

I_λ was obtained by combining (1), (2), and (3), as shown in Figure S1. The actinic flux during typical haze over Beijing (40ºN, 116ºE) on January 26, 2015 at 12:00 pm (GMT+8) was estimated using the National Center for Atmospheric Research Tropospheric Ultraviolet Visible (TUV) Radiation Model (Figure S1)[4, 5]. The input environmental parameters were set to be as follows: clouds optical depth = 0, base = 4, top = 5; aerosol optical depth = 2.3, single scattering albedo = 0.9, Angstrom exponent = 0.9; direct beam = diffuse down = diffuse up = 1. For clear days, the actinic flux was estimated over Beijing (at the same date and time) using the default parameters.

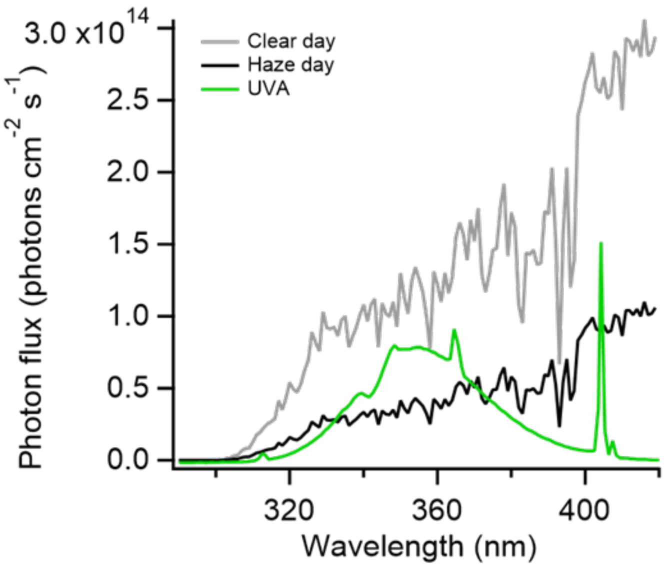

Figure S1. The photon flux in the flow cell and during typical haze days or clear days in Beijing, China.

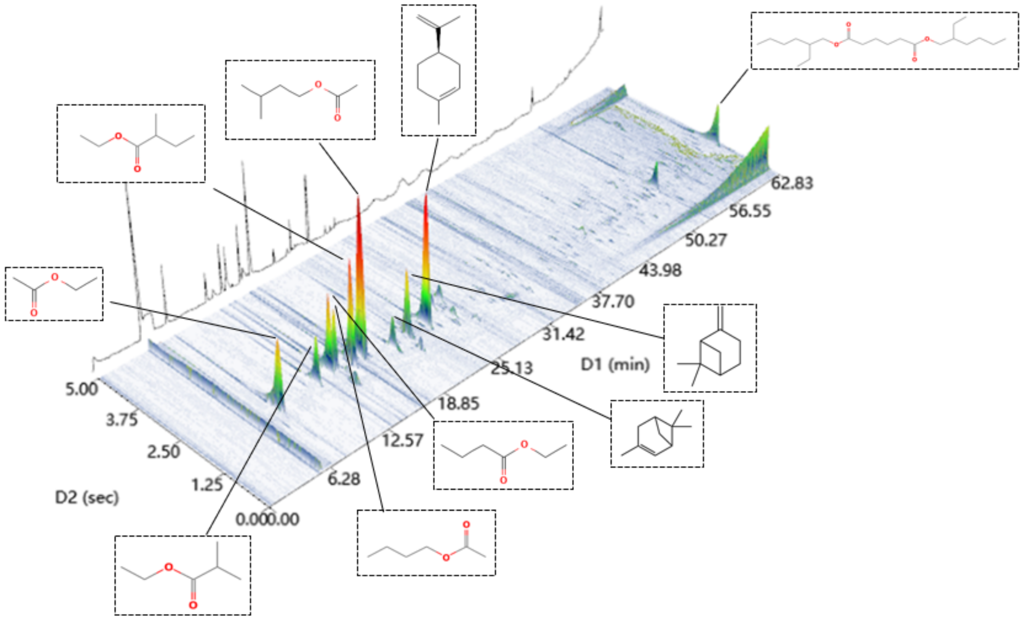

Figure S2. GC×GC chromatogram of VOC emissions from the VCP. The molecular structures of the top ten emitted species are shown.

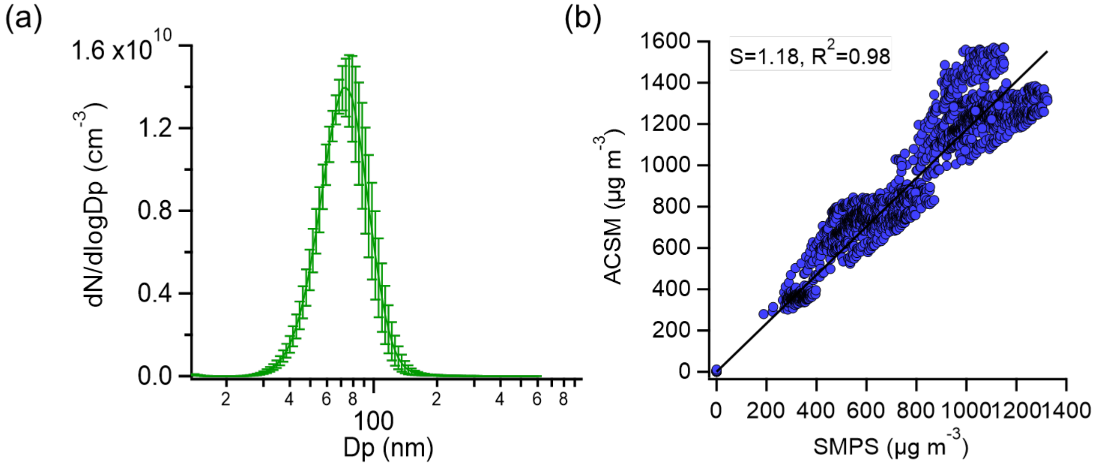

Figure S3. (a) The particle number size distribution of the VCP-derived SOA measured at the exit of the OFR and (b) scatter pot of total aerosol mass from ToF-ACSM measurements vs. mass estimated from SMPS measurements (assuming the aerosol density of 1 g cm^{-3}).

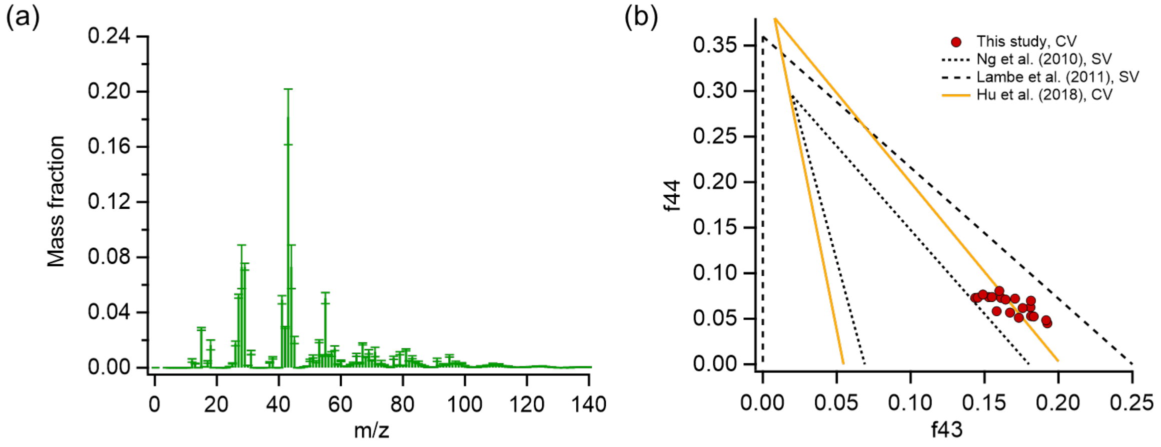

Figure S4 (a) The average mass spectra of VCP-derived SOA and (b) The fractions of the total organic signal at m/z 43 (f_{43}) vs. m/z 44 (f_{44}) from SOA data in this work. It also features triangle plots adapted from Ng et al.[6], Lambe et al.[7], and Hu et al.[8] The triangles help guide the eye to the regions where ambient oxygenated organic aerosol components typically fall. The legends “CV” and “SV” represent 'capture vaporizer' and 'standard vaporizer,' respectively, denoting the vaporizer used in the instruments.

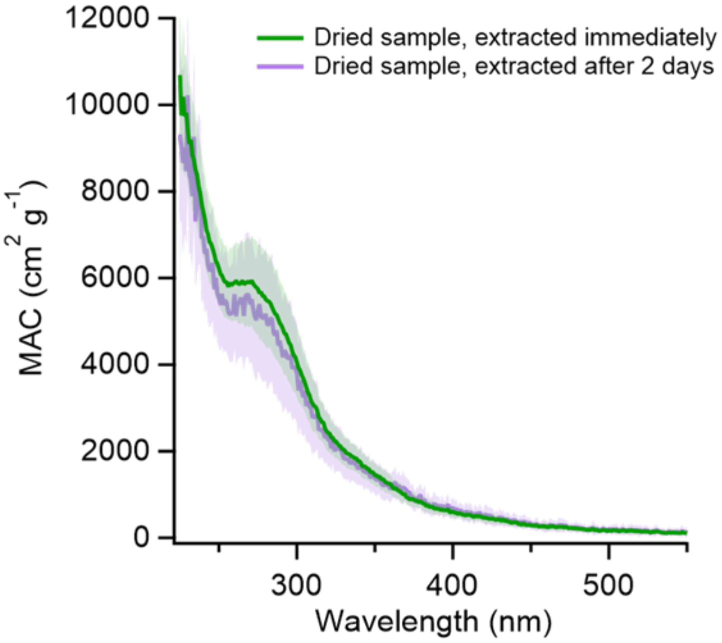

Figure S5. The absorption spectra of dried VCP-derived SOA filter samples extracted immediately (green) and 2 days after the collection (purple).

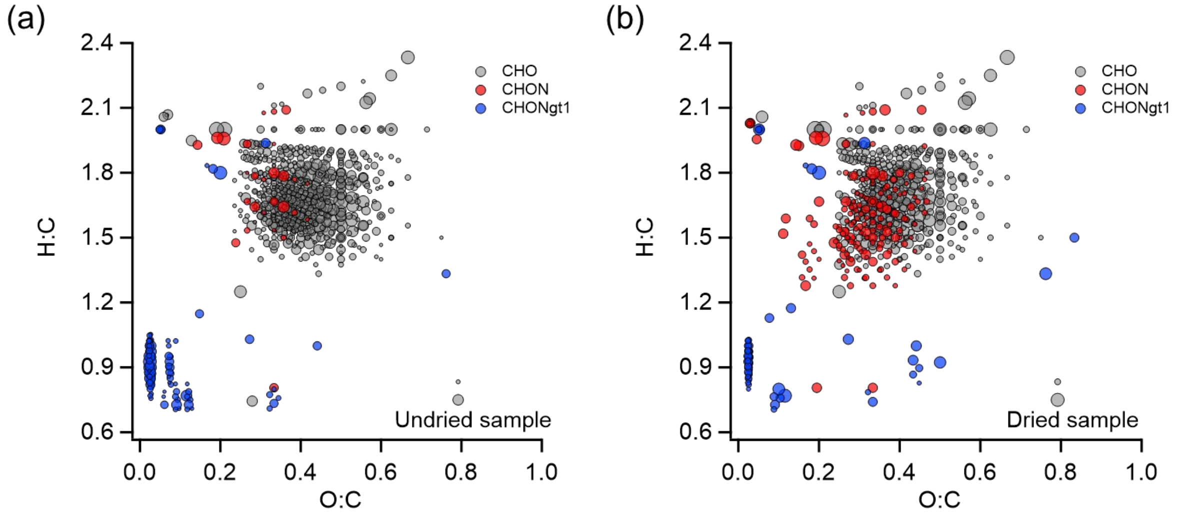

Figure S6. Van Krevelen plot of H:C vs. O:C obtained from assigned mass spectra of undried and dried VCP-derived SOA. Symbol size is proportional to the logarithmic relative intensity of corresponding peaks.

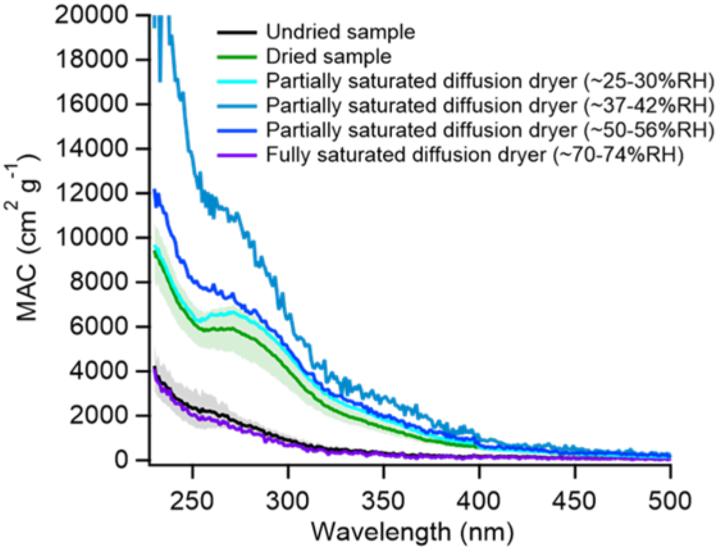

Figure S7. UV-Vis absorption spectra of undried and dried VCP-derived SOA and those dried at higher RH levels of ~25-30%, 37-42%, 50-56%, and 70-74% in the diffusion dryer (partially to almost fully saturated with water). The shaded regions represent the standard deviation (1σ).

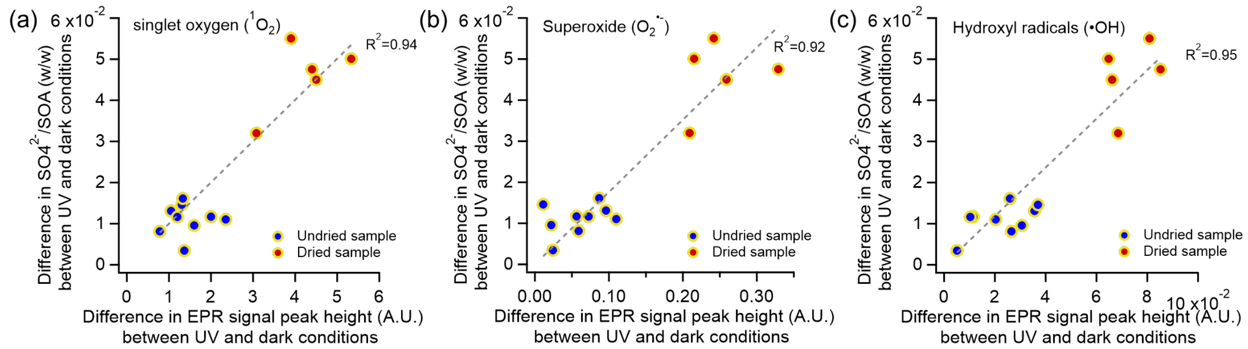

Figure S8. Relationship between the difference in sulfate production and the difference in EPR signal peak height under UV and dark conditions of (a) singlet oxygen ($^1O_2$), (b) superoxide ($O_2^{\bullet-}$), and (c) hydroxyl radical ($^\bullet OH$) in undried and dried VCP-derived SOA samples.

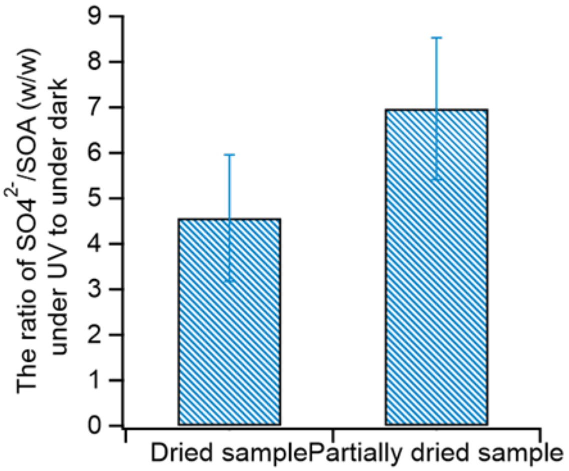

Figure S9. The normalized sulfate production ratio of UV to dark conditions for the dried sample and partially dried sample (~37-42% RH), after 10 h of reaction at 2 ppm SO_2 and 80% RH.

Table S1. Chemical profile identified in VOC emissions from the VCP[a].

compound	[1]D Retention time (min)	[2]D Retention time (s)	Peak area	Relative signal intensity %	CAS	Chemical formula	forward match degree	reverse match degree
1-Butanol, 3-methyl-, acetate	17.1739	1.821	1336394	20.315	123-92-2	C7H14O2	862	862
D-Limonene	23.924	1.651	1216642.5	18.495	5989-27-5	C10H16	870	870
Ethyl Acetate	7.924	1.751	527182.61	8.014	141-78-6	C4H8O2	811	865
Butanoic acid, 2-methyl-, ethyl ester	16.0074	1.82	523592.42	7.959	7452-79-1	C7H14O2	848	857
Butanoic acid, ethyl ester	13.924	1.913	469093.42	7.131	105-54-4	C6H12O2	863	863
Acetic acid, butyl ester	14.5073	1.923	368128.38	5.596	123-86-4	C6H12O2	826	827
Hexanedioic acid, bis(2-ethylhexyl) ester	62.3406	1.675	366233.25	5.567	103-23-1	C22H42O4	826	849
Bicyclo[3.1.1]heptane, 6,6-dimethyl-2-methylene-	21.8406	1.707	316292.08	4.808	127-91-3	C10H16	874	884
Propanoic acid, 2-methyl-, ethyl ester	12.2575	1.86	276864.56	4.209	97-62-1	C6H12O2	822	823
Bicyclo[3.1.1]hept-2-ene, 3,6,6-trimethyl-	19.8408	1.63	197772.29	3.006	4889-83-2	C10H16	853	865
n-Hexadecanoic acid	52.7574	1.507	145760.91	2.216	10/3/1957	C16H32O2	862	866
4-Acetyl-1-methylcyclohexene	27.0907	2.058	82640.251	1.256	9/1/6090	C9H14O	695	702
3-Cyclohexen-1-carboxaldehyde, 3,4-dimethyl-	25.9241	2.012	69724.024	1.06	0-0-0	C9H14O	732	739
3-Octanol, 3,7-dimethyl-	26.5074	1.527	48386.389	0.736	78-69-3	C10H22O	846	856
Hexanoic acid, ethyl ester	22.3407	1.772	31589.412	0.48	123-66-0	C8H16O2	813	868
Pentanoic acid, 2,2,4-trimethyl-3-carboxyisopropyl, isobutyl ester	43.0907	1.567	30123.16	0.458	0-0-0	C16H30O4	786	789
1,3-Dimethyl-1-cyclohexene	15.0906	1.727	28718.715	0.437	2808-76-6	C8H14	873	877
β-Myrcene	22.0074	1.644	26167.199	0.398	123-35-3	C10H16	806	877
7-Oxabicyclo[4.1.0]heptane, 1-methyl-4-(2-methyloxiranyl)-	33.5907	2.269	25137.873	0.382	8/2/1996	C10H16O2	765	831
1-Hexanol	16.8407	1.981	21973.053	0.334	111-27-3	C6H14O	802	848
1,2-Benzenedicarboxylic acid, bis(2-methylpropyl) ester	50.5073	2.241	20290.17	0.308	84-69-5	C16H22O4	840	850
3-Decen-1-ol, (Z)-	30.5905	1.633	18337.3	0.279	10340-22-4	C10H20O	791	848
Nonanal	26.7574	1.727	17735.554	0.27	124-19-6	C9H18O	741	831
Methyl anthranilate	35.514	2.706	17628.222	0.268	134-20-3	C8H9NO2	720	854
3-Hexen-1-ol, acetate, (Z)-	22.5907	1.904	17635.829	0.268	3681-71-8	C8H14O2	670	828
Ethanolamine	52.094	0.815	16753.216	0.255	141-43-5	C2H7NO	588	858
Octadecanoic acid	57.4242	1.527	16679.991	0.254	11/4/1957	C18H36O2	714	815

4-Methyl-5H-furan-2-one	21.4242	3.538	16577.41	0.252	6124-79-4	C5H6O2	685	828
Ethyl anthranilate	37.308	3.186	15042.062	0.229	87-25-2	C9H11NO2	790	846
Pentadecanal-	46.6741	1.411	13834.76	0.21	11/9/2765	C15H30O	748	912
Trimethylpyrazine	22.954	2.796	13063.351	0.199	14667-55-1	C7H10N2	692	877
1,1'-Biphenyl, 3,4-diethyl-	45.8409	2.277	12843.528	0.195	61141-66-0	C16H18	716	771
Benzophenone	44.674	2.852	11628.537	0.177	119-61-9	C13H10O	792	891
Hexadecanoic acid, methyl ester	52.0074	1.401	11117.465	0.169	112-39-0	C17H34O2	696	817
Tetradecanoic acid	47.5906	1.516	10981.248	0.167	544-63-8	C14H28O2	624	822
3-Heptanone, 6-methyl-	19.9241	1.846	10850.713	0.165	624-42-0	C8H16O	608	896
.psi.,.psi.-Carotene, 1,1',2,2'-tetrahydro-1,1'-dimethoxy-	31.5906	1.694	10812.457	0.164	13833-01-7	C42H64O2	526	560
Tetradecanal	43.7575	1.413	9782.0124	0.149	124-25-4	C14H28O	694	878
2,4-Di-tert-butylphenol	40.5908	1.858	9005.4804	0.137	96-76-4	C14H22O	789	893
2-Pentadecanone	46.1741	1.41	8909.4597	0.135	2345-28-0	C15H30O	636	796
Heptadecane	46.1741	1.095	8698.8962	0.132	629-78-7	C17H36	900	900
Dodecanal	37.5907	1.481	8698.7655	0.132	112-54-9	C12H24O	616	868
Heptane, 2,4-dimethyl-	14.8405	1.476	8153.2493	0.124	2213-23-2	C9H20	718	871
Ethanol, 2-(9-octadecenyloxy)-, (Z)-	49.3407	1.407	7820.8536	0.119	5353-25-3	C20H40O2	725	786
Dodecane	30.3405	1.295	7095.2898	0.108	112-40-3	C12H26	900	900
2-Hexenoic acid, 6-(2-methylenecyclopropyl)-, methyl ester	7.4239	1.508	6384.6355	0.097	0-0-0	C11H16O2	504	617
3-Dodecene, (Z)-	30.0073	1.293	6254.7612	0.095	7239-23-8	C12H24	554	864
1,2-Benzenedicarboxylic acid, butyl octyl ester	52.8406	2.326	5871.7133	0.089	84-78-6	C20H30O4	664	780
(Z)6-Pentadecen-1-ol	40.7573	1.455	5502.8242	0.084	68797-95-5	C15H30O	632	867
Undecane	26.5074	1.263	5383.7409	0.082	1120-21-4	C11H24	900	900
2-Heptadecanone	51.4241	1.438	5307.7914	0.081	2922-51-2	C17H34O	615	755
Naphthalene, 1,2,3-trimethyl-4-propenyl-, (E)-	46.6741	2.346	4821.0587	0.073	26137-53-1	C16H18	688	809
(5β)Pregnane-3,20β-diol, 14α,18α-[4-methyl-3-oxo-(1-oxa-4-azabutane-1,4-diyl)]-, diacetate	29.7573	1.325	4429.3046	0.067	0-0-0	C28H43NO6	540	575
E-10-Pentadecenol	34.174	1.501	4317.7055	0.066	0-0-0	C15H30O	634	826
1,1'-Biphenyl, 3,4-diethyl-	46.1741	2.243	4285.7403	0.065	61141-66-0	C16H18	652	746
Pentadecane	40.3408	1.122	3645.6743	0.055	629-62-9	C15H32	900	900

(5β)Pregnane-3,20β-diol, 14α,18α-[4-methyl-3-oxo-(1-oxa-4-azabutane-1,4-diyl)]-, diacetate	24.424	1.612	1278.8525	0.055	0-0-0	C28H43NO6	547	578
Ethyl iso-allocholate	49.8408	1.725	3625.6954	0.055	0-0-0	C26H44O5	595	653
Naphthalene, 1,2,3-trimethyl-4-propenyl-, (E)-	45.2573	2.266	3200.5936	0.049	26137-53-1	C16H18	635	817
Hexadecane, 1,1-bis(dodecyloxy)-	46.0073	1.144	3006.7143	0.046	56554-64-4	C40H82O2	596	706
10-Heneicosene (c,t)	40.174	1.152	2925.1975	0.044	95008-11-0	C21H42	563	851
17-(1,5-Dimethylhexyl)-10,13-dimethyl-3-styrylhexadecahydrocyclopenta[a]phenanthren-2-one	21.5906	2.204	2762.1564	0.042	0-0-0	C35H52O	526	554
Pentane, 2-methyl-	7.0907	1.403	2641.3332	0.04	107-83-5	C6H14	725	957
Octadecane	48.8407	1.108	2641.9464	0.04	593-45-3	C18H38	900	900
2H-Benzo[f]oxireno[2,3-E]benzofuran-8(9H)-one, 9-[[[2-(dimethylamino)ethyl]amino]methyl]octahydro-2,5a-dimethyl-	30.0073	1.669	2489.676	0.038	0-0-0	C19H32N2O3	535	696
Hexadecane	43.3407	1.095	2430.7475	0.037	544-76-3	C16H34	900	900
Benzeneethanol, α,α-dimethyl-, acetate	34.5908	1.959	2398.6245	0.036	151-05-3	C12H16O2	508	765
Akuammilan-17-ol, 10-methoxy-	11.4239	2.055	2276.3925	0.035	56259-10-0	C20H24N2O2	539	647
β-Carotene-3,3'-diol, (3R,3'R)-all-trans-	44.5908	1.07	2211.0307	0.034	144-68-3	C40H56O2	525	539
3-Pyridinecarboxylic acid, 2,7,10-tris(acetyloxy)-1,1a,2,3,4,6,7,10,11,11a-decahydro-1,1,3,6,9-pentamethyl-4-oxo-4a,7a-epoxy-5H-cyclopenta[a]cyclopropa[f]cycloundecen-11-yl ester, [1aR-(1aR*,2R*,3S*,4aR*,6S*,7S*,7aS*,8E,10R*,11R*,11aS*)]-	47.6739	2.028	2183.1185	0.033	51906-00-4	C32H39NO10	571	608

[a]Compounds with a relative signal intensity greater than 0.03% are shown.

References

(1) Liang, Z.; Zhou, L.; Infante Cuevas, R. A.; Li, X.; Cheng, C.; Li, M.; Tang, R.; Zhang, R.; Lee, P. K.; Lai, A. C., Sulfate Formation in Incense Burning Particles: A Single-Particle Mass Spectrometric Study. *Environmental Science & Technology Letters* **2022**.
(2) Mabato, B. R. G.; Lyu, Y.; Ji, Y.; Li, Y. J.; Huang, D. D.; Li, X.; Nah, T.; Lam, C. H.; Chan, C. K., Aqueous secondary organic aerosol formation from the direct photosensitized oxidation of vanillin in the absence and presence of ammonium nitrate. *Atmospheric Chemistry and Physics* **2022,** *22*, (1), 273-293.
(3) Galbavy, E. S.; Ram, K.; Anastasio, C., 2-Nitrobenzaldehyde as a chemical actinometer for solution and ice photochemistry. *Journal of Photochemistry and Photobiology A: Chemistry* **2010,** *209*, (2-3), 186-192.
(4) Che, H.; Xia, X.; Zhu, J.; Wang, H.; Wang, Y.; Sun, J.; Zhang, X.; Shi, G., Aerosol optical properties under the condition of heavy haze over an urban site of Beijing, China. *Environmental Science and Pollution Research* **2015,** *22*, (2), 1043-1053.
(5) Che, H.; Xia, X.; Zhu, J.; Li, Z.; Dubovik, O.; Holben, B.; Goloub, P.; Chen, H.; Estelles, V.; Cuevas-Agulló, E., Column aerosol optical properties and aerosol radiative forcing during a serious haze-fog month over North China Plain in 2013 based on ground-based sunphotometer measurements. *Atmospheric Chemistry and Physics* **2014,** *14*, (4), 2125-2138.
(6) Ng, N.; Canagaratna, M.; Zhang, Q.; Jimenez, J.; Tian, J.; Ulbrich, I.; Kroll, J.; Docherty, K.; Chhabra, P.; Bahreini, R., Organic aerosol components observed in Northern Hemispheric datasets from Aerosol Mass Spectrometry. *Atmospheric Chemistry and Physics* **2010,** *10*, (10), 4625-4641.
(7) Lambe, A.; Onasch, T.; Massoli, P.; Croasdale, D.; Wright, J.; Ahern, A.; Williams, L.; Worsnop, D.; Brune, W.; Davidovits, P., Laboratory studies of the chemical composition and cloud condensation nuclei (CCN) activity of secondary organic aerosol (SOA) and oxidized primary organic aerosol (OPOA). *Atmospheric Chemistry and Physics* **2011,** *11*, (17), 8913-8928.
(8) Hu, W.; Day, D. A.; Campuzano-Jost, P.; Nault, B. A.; Park, T.; Lee, T.; Croteau, P.; Canagaratna, M. R.; Jayne, J. T.; Worsnop, D. R., Evaluation of the new capture vaporizer for aerosol mass spectrometers: Characterization of organic aerosol mass spectra. *Aerosol Science and Technology* **2018,** *52*, (7), 725-739.
